# Characterization and Emulsification Properties of Rhamnolipid and Sophorolipid Biosurfactants and Their Applications

**DOI:** 10.3390/ijms12021232

**Published:** 2011-02-18

**Authors:** Thu T. Nguyen, David A. Sabatini

**Affiliations:** 1 Department of Chemical Engineering, University of Utah, 50 S Central Campus Drive, MEB 3290, Salt Lake City, UT 84112, USA; 2 Department of Civil Engineering and Environmental Sciences, Institute for Applied Surfactant Research, University of Oklahoma, 202 W. Boyd, CEC 334, Norman, OK 73019, USA; E-Mail: sabatini@ou.edu

**Keywords:** rhamnolipid biosurfactant, sophorolipid biosurfactant, characterization, microemulsions, application

## Abstract

Due to their non-toxic nature, biodegradability and production from renewable resources, research has shown an increasing interest in the use of biosurfactants in a wide variety of applications. This paper reviews the characterization of rhamnolipid and sophorolipid biosurfactants based on their hydrophilicity/hydrophobicity and their ability to form microemulsions with a range of oils without additives. The use of the biosurfactants in applications such as detergency and vegetable oil extraction for biodiesel application is also discussed. Rhamnolipid was found to be a hydrophilic surfactant while sophorolipid was found to be very hydrophobic. Therefore, rhamnolipid and sophorolipid biosurfactants in mixtures showed robust performance in these applications.

## 1. Introduction

**Figure 1.**
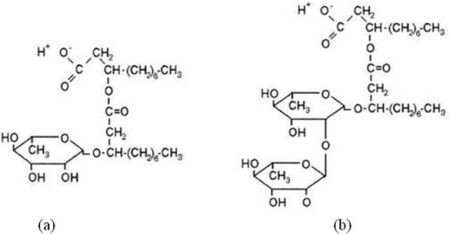
Structures of the rhamnolipids: **(a)** monorhamnolipid and **(b)** dirhamnolipid (adapted from [1]).

**Figure 2.**
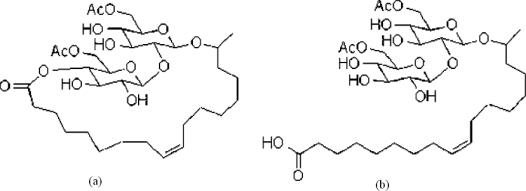
Structures of the sophorolipids (Ac = Acetyl): **(a)** lactone form and **(b)** acidic form (adapted from [2]).

Rhamnolipid (Figure 1) and sophorolipid (Figure 2) biosurfactants are glycolipid biosurfactants which are generally composed of carbohydrate heads and lipid tails [1,2]. Rhamnolipid biosurfactants discussed in this review were produced by *Pseudomonas aeruginosa* growing on glucose. They have two hydrophilic head groups: the carboxylate group that gives the rhamnolipids an anionic character and the rhamnosyl that contributes to the bulkiness of the head group. They have two identical tails of C8 alkyl chain [3,4]. On the other hand, sophorolipid biosurfactants have only one long tail of an unsaturated fatty acid [2]. There are often two conformations of the sophorolipids during production: the lactone form resulting from the esterification of the carboxylic acid group to the disaccharide ring (Figure 2a) and the acidic form with two head groups of dimeric sugar sophorose and carboxylic acid (Figure 2b), in which the sophorose head is acetylated [1]. This review focuses on the lactone acetylated sophorolipid biosurfactants, produced by *Candida bombicola* growing on a mixture of glucose and fatty acids, specifically palmitic and oleic acids. It should be noted that rhamnolipid and sophorolipid biosurfactants produced by other microorganisms growing on different substrates can have different molecular structures and compositions. The molecular structures suggest that the rhamnolipid is a hydrophilic surfactant while the sophorolipid is a hydrophobic surfactant. This article reviews the technique to characterize the hydrophilicity/hydrophobicity of these two biosurfactants and the evaluation of their microemulsion formation for a range of oils.

**Figure 3.**
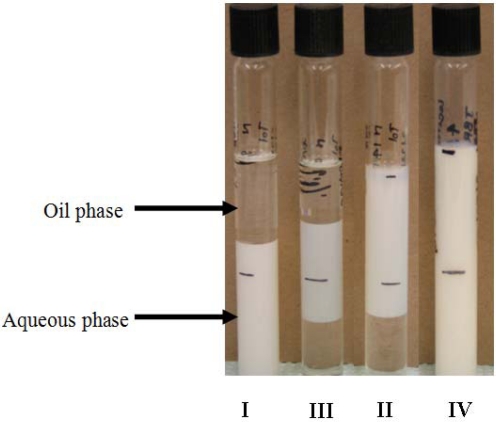
Four types of Winsor microemulsions.

Microemulsions are thermodynamically stable dispersion of two immiscible liquids (oil and water) stabilized by surfactant films [5]. Microemulsions can exist in four forms (Figure 3), known as Winsor type microemulsions [5,6]. Type I (oil-in-water or O/W) microemulsions solubilize oil in spherical normal micelles within the water-continuous phase while Type II (water-in-oil or W/O) microemulsions solubilize water in reverse micelles within the oil-continuous phase. Type III microemulsions are three-phase systems in which the middle phase microemulsions are in equilibrium with both excess oil and excess water phases. Type IV microemulsions are the expansion of the middle phase microemulsions at high surfactant concentration such that all the excess oil and excess water are incorporated into a single phase.

Rhamnolipid and sophorolipid biosurfactants have been evaluated for uses in many applications such as bioremediation, microbial enhanced oil recovery, food and cosmetic industries and pharmaceutical applications [7–12]. In this paper, the use of rhamnolipid and sophorolipid biosurfactants in detergency and vegetable oil extraction for biodiesel application is exclusively reviewed.

## 2. Surfactant Characterization

### 2.1. Characteristic Curvature and Rhamnolipid Characterization

The characteristic curvature (Cc) of a surfactant was proposed by Acosta *et al.* [13] as the surfactant parameter that reflects the tendency of the surfactant to form normal micelles, reverse micelles or intermediate aggregates. The value of the characteristic curvature ranges from negative to positive with negative values for hydrophilic surfactants and positive values for hydrophobic surfactants. Therefore, a surfactant with negative Cc value tends to form O/W microemulsions (normal micelles) while a surfactant with positive Cc value tends to form W/O microemulsions (reverse micelles). This paper reviews the characterization of rhamnolipid biosurfactants by determining the Cc value of the surfactant.

To determine the Cc value of a surfactant, it is useful to introduce the hydrophilic-lipophilic deviation (HLD) concept proposed by Salager *et al.* [14,15]. Since rhamnolipid is an anionic surfactant, the HLD equation for ionic surfactants is introduced in this paper [15]:

(1) $$\text{HLD}=\text{ln}(S)-K\times N_{C,O}-f(A)+\sigma -\alpha_{T}\Delta T$$

where *S* is the salinity in the aqueous phase (g/100 mL); *N_C,O_* is the equivalent alkane carbon number of the oil; *f(A)* is the function of the type and concentration of the alcohol used, if there is no alcohol in the formulation, *f(A)* = 0; *σ* is the surfactant parameter which was redefined by Acosta *et al.* [13] as the characteristic curvature Cc; and Δ*T* is the difference between the experimental temperature and the reference temperature, which is 25 °C.

As the HLD value equals 0, the surfactant is equally soluble in oil and water and middle phase bicontinuous microemulsions (Type III or Type IV) are formed. A negative value of HLD indicates a hydrophilic surfactant system and O/W microemulsions (Type I) are formed while a positive value of HLD indicates a hydrophobic surfactant system and W/O microemulsions (Type II) are formed [13]. Therefore, as HLD = 0, the formulation is at optimum and *S* in Equation 1 is denoted as S^*^, optimum salinity. Based on this concept, Acosta *et al.* [13] developed an equation to estimate the Cc value of a target surfactant in mixtures with a reference surfactant with a known Cc value:

(2) $$\text{ln}\;(S^{*}/S_{1}^{*})=X_{2}[({\text{Cc}}_{1}-{\text{Cc}}_{2})+({\text{K}}_{2}-{\text{K}}_{1})\;N_{C,O}]$$

where S^*^ is the optimum salinity of the surfactant mixture, S_1_^*^ is the salinity of the reference surfactant, *X_2_* is the molar fraction of the target surfactant in the mixture and 1 and 2 denotes for the reference and the target surfactants, respectively. When the oil used for microemulsion formation is benzene, *N_C,O_* = 0 and Equation 2 can be simplified as:

(3) $$\text{ln}\;(S^{*}/S_{1}^{*})=X_{2}({\text{Cc}}_{1}-{\text{Cc}}_{2})$$

**Figure 4.**
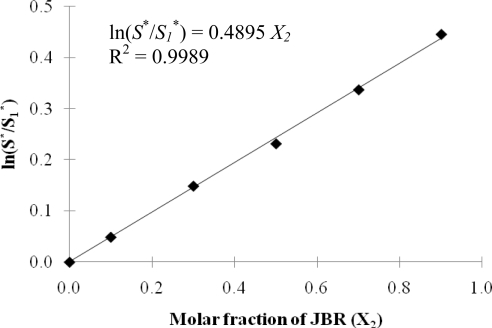
Shift in optimum electrolyte concentration [ln (S^*^/S_1_^*^)] for SDHS-JBR-benzene at 23 ± 1 °C microemulsions as a function of the fraction of JBR in the system (Reprinted from [16]. Reprinted with permission from SpringerLink).

Nguyen and Sabatini (16) quantitatively characterized the hydrophilicity/hydrophobicity of rhamnolipid (JBR) biosurfactant using these concepts. In their work, sodium dihexyl sulfosuccinate (SDHS) was used as the reference surfactant with a Cc value of −0.92 [13]. Phase behavior studies were performed for mixtures of JBR and SDHS at different surfactant ratios at room temperature to determine optimum salinity values. With benzene used as the oil, a correlation presented in Equation 3 between optimum salinity and molar fraction of JBR in the surfactant mixture was found as shown on Figure 4.

From the plot in Figure 4 and the correlation in Equation 3, the value of (Cc_1_ – Cc_2_) was detemined as the slope with a value of 0.4895. Thus, knowing the Cc_1_ value of −0.92 for SDHS, the value of Cc_2_ for JBR was calculated to be −1.41. The negative value of Cc_2_ indicates that rhamnolipid is a hydrophilic surfactant and the magnitude of Cc_2_ indicates that rhamnolipid (−1.41) is more hydrophilic that SDHS (−0.92). This result is consistent with the finding from Nguyen *et al.* [17] where this trend was observed experimentally. Knowing the Cc value of rhamnolipid biosurfactant, one can quantitatively compare its hydrophilicity/hydrophobicity with conventional synthetic surfactants; examples are shown in Table 1. This can serve as a helpful guideline in replacing conventional synthetic surfactants with rhamnolipid in microemulsion formulation.

### 2.2. Winsor R-ratio, Optimum Salinity and Sophorolipid Characterization

The hydrophilicity/hydrophobicity of a surfactant can be evaluated using Winsor R-ratio concept, which is defined as:

(4) $$R=\frac{A_{\text{CO}}}{A_{\text{CW}}}$$

where *A_CO_* and *A_CW_* indicate the overall interaction between the surfactant adsorbed at the interface with the oil and the water, respectively [18]. As R < 1, the interaction between the surfactant and the oil phase (*A_CO_*) is smaller than the interaction between the surfactant and the water phase (*A_CW_*) and vice verse as R > 1. Therefore, for systems with R < 1, Type I microemulsions are formed and for systems with R > 1, Type II microemulsions are formed. At R = 1, the surfactant–water interaction and surfactant–oil interaction are balanced, resulting in the formation of Type III middle phase microemulsions. At this point, the formulation is at optimum where equal amounts of oil and water are solubilized in the middle phase microemulsion [19]. These water–surfactant–oil interactions can be manipulated by a change in a tuning parameter such as salinity or electrolyte concentration for ionic surfactants and temperature for nonionic surfactants. For example, for an ionic surfactant system, increasing salinity increases the interaction between the surfactant and the oil and decreases the interaction between the surfactant and the water. In other words, *A_CO_* increases and *A_CW_* decreases. As a result, the Winsor R ratio increases to 1; thus, a phase transition from Type I to Type III may occur. A further increase in salinity makes *A_CO_* become greater while *A_CW_* become smaller; the R ratio increases to greater than 1 and Type III to II transition may occur.

**Figure 5.**
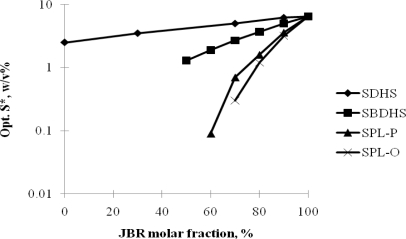
Optimum salinity (S^*^) for varying fraction of rhamnolipid in mixtures with SDHS (♦), SDBHS (▪), SPL-P (▴) and SPL-O (×) in microemulsion formulation with benzene. Total surfactant concentration is kept constant at 0.1 M for all mixtures and surfactant ratios (Reprinted from [20]. Reprinted with permission from Elsevier).

Based on the Winsor R-ratio concept, Nguyen *et al.* [20] evaluated the hydrophilicity/hydrophobicity of sophorolipid biosurfactants using the optimum salinity. In their work, salinity was varied in the phase study of sophorolipid/rhamnolipid biosurfactant mixtures to identify the optimum salinity (the salinity at which the system is within the Type III region and evidences a minimum in IFT) which is an indicator of the system hydrophobicity. For example, a lower value of optimum salinity indicates a more hydrophobic surfactant system to begin with since less salinity is required to move the surfactant to the optimum middle phase system. Benzene was used as the oil phase. The same experiments were done for two synthetic surfactants, sodium dihexyl sulfosuccinate (SDHS) and sodium bis(2-ethyl) dihexyl sulfosuccinate (SBDHS) for comparison since the hydrophilicity/hydrophobicity of the two surfactants was quantified. For each surfactant mixture with rhamnolipid biosurfactant (JBR), the optimum salinity was identified and plotted *versus* the molar fraction of JBR, which is the common surfactant in all mixtures, as shown in Figure 5. As can be seen in Figure 5, two kinds of lactone acetylated sophorolipid biosurfactants were studied, SPL-P and SPL-O synthesized by *Candida bombicola* from palmitic C16 fatty acid and oleic C18 fatty acid, respectively. Therefore, SPL-P has unsaturated C16 in the tail and SPL-O has unsaturated C18.

The results show that for all four studied surfactant mixtures, the optimum salinity increases with increasing JBR molar fraction and is highest at 100% JBR in mixtures, suggesting that rhamnolipid biosurfactant is more hydrophilic than all four studied surfactants. For all surfactant mixtures, increasing the molar fraction of JBR in the mixture increases the optimum salinity. At a certain JBR molar fraction, the optimum salinity decreases for the order of mixtures with SDHS > SBDHS > SPL-P > SPL-O. Based on Winsor R-ratio concept explained above, the hydrophobicity increases in this order for the four studied surfactant since the most hydrophobic surfactant results in the highest optimum salinity. SPL-P and SPL-O have very similar optimum salinity, except for that SPL-O is slightly more hydrophobic.

## 3. Applications

**Figure 6.**
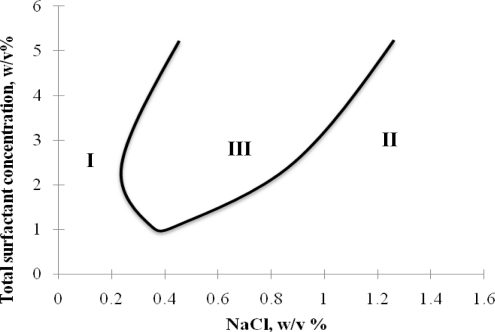
Partial fish phase diagram with diesel of biosurfactant mixtures: Lecithin/Sophorolipid/Rhamnolipid = 1/1/0.628 by weight ratio (Reprinted from [21]. Reprinted with permission from Elsevier).

**Figure 7.**
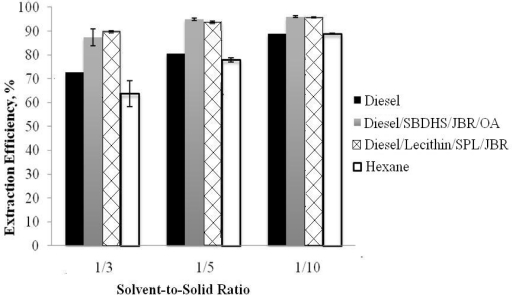
Effect of extraction solvent on oil extraction efficiency at 60 minute extraction time and 200 rpm shaking speed (SBDHS: sodium bis(2-ethyl) dihexyl sulfosuccinate, JBR: rhamnolipid, OA: oleyl alcohol, SPL: sophorolipid) (Reprinted from [21]. Reprinted with permission from Elsevier).

Biosurfactant mixtures were used in vegetable oil extraction for biofuel application by Nguyen and co-workers [21]. In this work, reverse-micellar microemulsions of diesel were used as the extraction solvent for vegetable oil extraction. Thus, the phase behavior with diesel using biosurfactant mixtures composed of lecithin, rhamnolipid and sophorolipid biosurfactants was studied (Figure 6). Three types of microemulsions were observed for the biosurfactant mixture with diesel at relatively low salinity. However, only Type II microemulsions or reverse-micellar microemulsions are of interest for the vegetable oil extraction process since this type of microemulsion has the oil (diesel in this case) as the continuous phase which extracts the vegetable oil trapped in the seeds based on the “like-dissolves-like” principle. Diesel-based reverse-micellar microemulsions of Lecithin/Sophorolipid/Rhamnolipid were shown to extract vegetable oil more effectively than diesel itself and even conventional hexane, as seen in Figure 7.

The concentration of Lecithin/Sophorolipid/Rhamnolipid used in this extraction was 0.5/0.5/0.31 wt%. Compared to the other surfactant mixture of SBHDS/Rhamnolipid/Oleyl alcohol at 0.56/0.72/0.34 wt%, the reverse-micellar microemulsions of the biosurfactant mixture achieved very similar extraction efficiency at a slightly lower total surfactant concentration. As can be seen in Figure 7, at the same extraction condition (60 minute and 200 rpm), higher extraction efficiency was obtained with diesel-based reverse micellar microemulsions of biosurfactant mixtures than with either diesel itself or conventional hexane.

**Figure 8.**
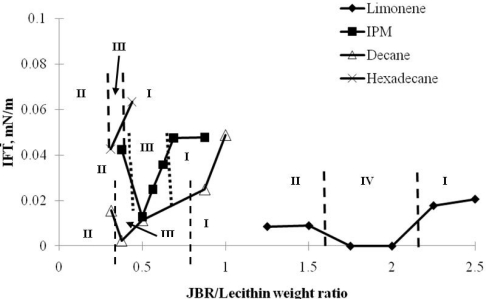
Interfacial tension and microemulsion for four different oils: limonene (♦), IPM (▪), decane (Δ) and hexadecane (×). Formulations were prepared with Lecithin/SPL concentration of 4/4% w/v and 0.9% w/v NaCl (Reprinted from [20]. Reprinted with permission from Elsevier).

The same biosurfactant mixture (Lecithin/Sophorolipid/Rhamnolipid) was also studied by Nguyen *et al.* [20] in microemulsion formations for a range of oil types and oil hydrophobicity. These oils include polar oils such as limonene and isopropyl myristate (IPM) and nonpolar oil such as decane and hexadecane. These four oils also demonstrate a wide range of oil hydrophobicity or EACNS (equivalent alkane carbon number) from 5.9 for limonene to 16 for hexadecane with oil of higher EACN being more hydrophobic. The results show the robust performance of the biosurfactant mixture in microemulsion formation and interfacial tension (IFT) reduction for all these oils (Figure 8). Middle phase microemulsions were formed for all four oils with ultralow IFT values.

**Figure 9.**
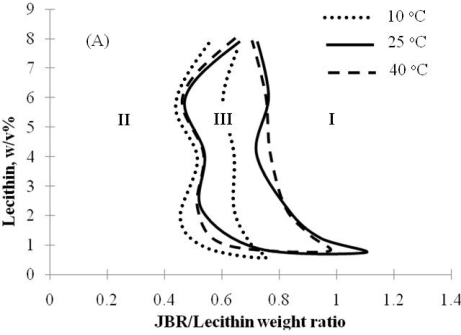

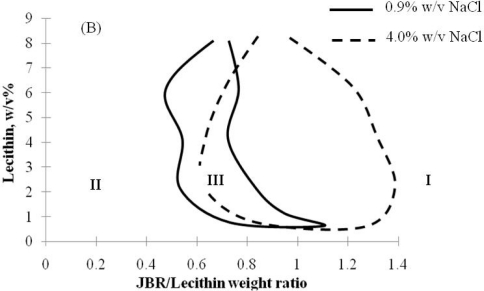
Phase behavior diagrams for biocompatible IPM-based microemulsions at different formulation conditions: **(A)** Effect of temperature and **(B)** Effect of electrolyte concentration. (Reprinted from [20]. Reprinted with permission from Elsevier).

Microemulsions for limonene and isopropyl myristate were studied in fish phase behavior diagrams (Figures 8 and 9) as potential applications for hard surface cleansers, cosmetics and pharmaceuticals. The effects of temperature and electrolyte concentration were evaluated on the phase behavior of IPM microemulsion since changes in temperature and electrolyte concentration are important considerations in microemulsions for cosmetics and drug delivery. It was found that IPM microemulsions using biosurfactant mixtures of Lecithin/Sophorolipid/Rhamnolipid were virtually temperature-insensitive and not significantly affected by the change in electrolyte concentration (Figure 9). These properties make biosurfactant formulations desirable in cosmetics and drug delivery applications.

**Figure 10.**
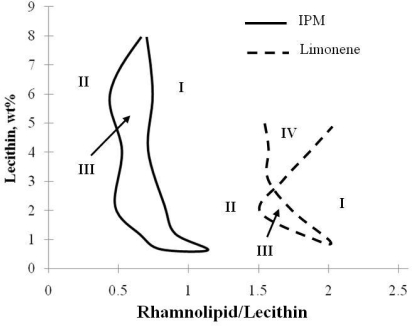
Phase behavior diagrams for IPM and Limonene microemulsions at 25 °C. Lecithin/Sophorolipid weight ratio = 1/1, 0.9 % w/v NaCl. (Reprinted from [20]. Reprinted with permission from Elsevier).

A comparison of IPM microemulsions and limonene microemulsions was made in Figure 10 to show how different oil requires different composition of the biosurfactant mixture to form middle phase microemulsions. It can be seen that limonene, the less hydrophobic oil, requires a more hydrophilic formulation (higher ratio of Rhamnolipid/Lecithin) to form middle phase (Type III) microemulsion and requires less total surfactant concentration to form single phase (Type IV) microemulsion.

**Figure 11.**
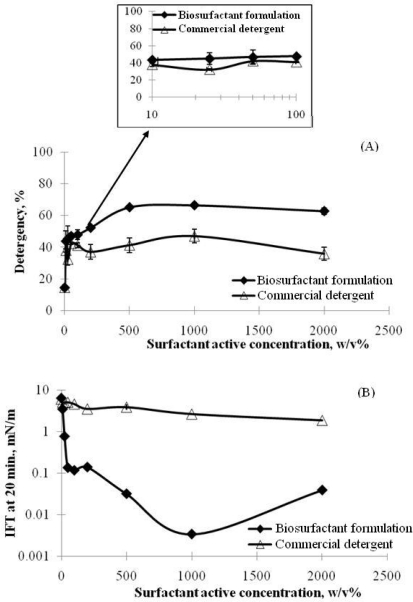
Detergency performance **(A)** and dynamic IFT **(B)** of the biosurfactant formulation vs. commercial detergent at different total surfactant active concentration. The biosurfactant formulation has Lecithin/SPL/JBR = 1.0/1.0/0.3 by wt. ratio and 0.9% w/v NaCl (Reprinted from [20]. Reprinted with permission from Elsevier).

Detergency tests were also performed for the biosurfactant formulation (Lecithin/Sophorolipid/Rhamnolipid) to investigate the detergency power of the formulation for hexadecane removal (Figure 10). The detergency performance was found to increase as the total active surfactant concentration increased for both the biosurfactant formulation and the commercial detergent (Figure 11A) while the dynamic IFT values were observed otherwise (Figure 11B). This inverse relationship between detergency performance and IFT values has also been observed in other detergency tests [22,23]. However, the biosurfactant formulation produces better cleaning efficiency as compared to the commercial detergent within the studied range of total active concentration. It also has comparable cleaning performance to the detergency tests done by Tongcumpou *et al.* [23] at much lower total active surfactant concentration. For example, the same detergency power (∼ 65%) was achieved at 0.1% w/v total active concentration and 0.9% w/v electrolyte concentration with the biosurfactant formulation *versus* at 0.25% w/v total active concentration and 5% w/v electrolyte concentration with the formulation of Tongcumpou *et al.* [23].

## 4. Conclusions

This review presents the methodology to characterize the surfactant properties of rhamnolipid and sophorolipid biosurfactants. It was found that rhamnolipid biosurfactant is relatively hydrophilic while sophorolipid biosurfactant is hydrophobic as compared to other synthetic surfactants. The biosurfactants in mixtures were able to produce microemulsions for a wide range of oils that are applicable for vegetable oil extraction for biofuel application, hard surface cleansers, drug delivery and detergents.

## Figures and Tables

**Table 1.** Characteristic curvature of conventional synthetic surfactants.

**Surfactants**	**Characteristic curvature (Cc)**
Sodium dodecyl sulfate (SDS)^a^	−2.34
Sodium octanoate^a^	−2.11
Rhamnolipid biosurfactant^b^	−1.41
Sodium dihexyl sulfosuccinate (SDHS)^a^	−0.92
Sodium dodecyl benzene sulfonate (SDBS)^a^	−0.91

a Cc values reported in [13];

b Cc value reported in [16].
